# Number of Prescription Medications and Overall Survival in Metastatic Castrate-Resistant Prostate Cancer

**DOI:** 10.1155/proc/6863066

**Published:** 2024-12-18

**Authors:** Carley R. Pickett, Daniel B. Eaton, Krishny Karunanandaa, Emily Cybulla, Brendan T. Heiden, Su-Hsin Chang, Yan Yan, Melanie P. Subramanian, Varun Puri, Martin W. Schoen

**Affiliations:** ^1^Division of Hematology and Medical Oncology, Department of Internal Medicine, Saint Louis University School of Medicine, St. Louis, Missouri, USA; ^2^Division of Hematology and Medical Oncology, Department of Internal Medicine, Veterans Affairs St. Louis Health Care System, St. Louis, Missouri, USA; ^3^Division of Cardiothoracic Surgery, Department of Surgery, Washington University School of Medicine, St. Louis, Missouri, USA; ^4^Division of Public Health Sciences, Department of Surgery, Washington University School of Medicine, St. Louis, Missouri, USA

**Keywords:** Charlson Comorbidity Index, comorbidity, medications, mortality, prostate cancer, Veterans Health

## Abstract

**Background:** Assessment of comorbid diseases is essential to clinical research and may risk-stratify patients for mortality independent of established methods such as the Charlson Comorbidity Index (CCI).

**Methods:** In a retrospective study of U.S. Veterans, we examined the association between the number of medications, 1-year mortality, and overall survival in Veterans being treated for metastatic castration-resistant prostate cancer (mCRPC) between 2011 and 2017.

**Results:** Among 8855 Veterans, a median of 11 medications and 6 medication classes were filled in the year prior to initial treatment of mCRPC with abiraterone or enzalutamide. The median patient age was 74 years, 25.7% of patients were Black, and the median CCI was 3. Despite being associated with fewer medications, increasing age was associated with an increased CCI. After adjusting for patient, tumor, and treatment factors, both the number of medications and the number of medication classes were associated with increased 1-year mortality with adjusted OR (95% CI) of 1.03 (1.03, 1.04) and 1.08 (1.06, 1.11), respectively. Medications within Anatomic Therapeutic Class (ATC) N (nervous system) and ATC G (genitourinary and sex hormones) were associated with decreased OS, HR 1.18 (1.11, 1.25) and HR 1.15 (1.10, 1.20), respectively. Medications within ATC C (cardiovascular) were associated with increased OS, HR 0.91 (0.86, 0.97). Within a subgroup of patients with comparable age and CCI, the increased number of medications was associated with the increased risk of death.

**Conclusions:** The number and type of medications were independently associated with survival in patients undergoing treatment for mCRPC. With new therapies for treatment of advanced prostate cancer, patients are living longer, which increases the need for better understanding of the impact of comorbid diseases. Simple methods to assess disease burden and prognosticate survival have the potential to guide treatment decisions and improve the quality of life in this patient population.

## 1. Introduction

Prostate cancer is the most common cancer among men, and despite having generally favorable outcomes, up to one-third of patients will develop metastatic disease [1]. With the availability of new therapies for the treatment of advanced prostate cancer, patients are living longer with a 32% 5-year survival [2–5]. Regardless of the treatment regimen, both age and comorbid diseases are significant predictors in determining the risk of death, even in metastatic prostate cancer [6]. By identifying and understanding the impact of comorbid diseases, clinicians may prevent adverse events and improve the survivorship experience of this patient population.

Several comorbidity assessment tools that use administrative billing codes have been developed to help predict long-term prognosis and survival. Two of the most widely used and validated indices are the Charlson Comorbidity Index (CCI) [7] and the Elixhauser Comorbidity Index [8]. The CCI is a weighted index based on 19 International Classification of Diseases (ICD) diagnosis codes that predicts 10-year survival. Elixhauser is an unweighted system that uses ICD codes to predict the length of hospital stay, hospital charges, and in-hospital mortality. However, studies have shown several flaws with these indices, including underreporting or failure to code for comorbidities in administrative data [9, 10] and variation in ICD codes for the same diagnosis [11, 12]. This indicates that ICD codes are often too broad and not an accurate representation of the severity of an illness. Finally, the calculation of a comorbidity index is not currently available in electronic health records, making it impractical to perform in an individual patient encounter, necessitating alternative approaches that can be implemented in routine clinical practice.

As a result, alternative systems to assess comorbid diseases have been developed. Given the assumption that pharmaceuticals are used to treat specific conditions, novel methods have used patient medications to determine comorbid diseases. An initial system, called the Chronic Disease Score, used automated pharmacy data to predict hospitalizations and mortality [13]. This scoring system has been updated and renamed the Rx-Risk Comorbidity Index [14] and validated in a population of Veterans [15]. Additional drug-based indices have also been utilized for a range of chronic medical conditions [15–19]. When comparing prescription medications with CCI, one study found that 73% of patients were categorized as free of comorbidity according to the CCI, but 84% had received at least one prescription [20]. This raises the question of which index is optimal for defining comorbid diseases.

Comorbid illness can influence survival and treatment decisions in patients with metastatic prostate cancer [6], making it important to accurately determine the number and severity of comorbid diseases. The aim of our study is to examine how the number and class of prescription medications predict survival in patients with metastatic castrate-resistant prostate cancer (mCRPC) in comparison with the established methods, such as CCI.

## 2. Material and Methods

### 2.1. Data and Patient Population

The Veterans Health Administration (VHA) Informatics and Computing Infrastructure (VINCI) was used to access the Corporate Data Warehouse to identify a nationwide cohort of Veterans with mCRPC. All Veterans treated with abiraterone or enzalutamide for mCRPC from May 2011 to June 2017 were included in the study. Any Veterans without covariates used in the analysis were excluded. Any Veterans with zero dispensed prescription medications were excluded to limit missing data given the assumption that these Veterans were receiving care from outside the Veterans Affairs (VA) health system. Additionally, medications that were prescribed, but not dispensed by the pharmacy were excluded from the study to limit concerns regarding medication compliance. Patients were followed until April 2020—the date of censoring. This study was reviewed by the Saint Louis Veterans Affairs Medical Center Institutional Review Board and was performed in accordance with the Declaration of Helsinki. A waiver of consent was approved given the retrospective nature of the analyses. The results were reported according to the Strengthening the Reporting of Observational Studies in Epidemiology (STROBE) guidelines.

### 2.2. Outcomes and Covariates

Prescription medications were obtained using the Corporate Data Warehouse Pharmacy Outpatient file. All medications dispensed in the past year prior to the start of treatment with abiraterone or enzalutamide were collected. Time zero was the start of treatment with abiraterone or enzalutamide regardless of the type of prior prostate cancer treatment the Veteran had received. The number of unique medications was determined based on the Anatomical Therapeutic Chemical (ATC) classification system, which is characterized into 14 main anatomical or pharmacological groups. Additional covariates from the VHA Corporate Data Warehouse including age, CCI, body mass index (BMI), prostate-specific antigen (PSA), race (White vs. Black), and prior docetaxel use were collected at or value immediately before first prescription of enzalutamide and abiraterone. All Veterans without this data were not included in the analysis. The Romano and Quan adaptation of the CCI were calculated based on ICD-9/10 codes obtained any time prior to treatment initiation with the exclusion of prostate cancer [9, 21]. The primary outcome of this study was overall survival, which was determined from the date of initial treatment to death or censoring in April 2020. We assessed a secondary outcome of 1-year mortality. Death was ascertained from the VA vital status file.

### 2.3. Statistical Analysis

Demographic and clinical characteristics were compared between patients using chi-square, Student's *t*, Wilcoxon two-sample, or Kaplan–Meier curves with log-rank tests as appropriate for unadjusted analyses. Multivariable logistic regression and Cox proportional hazard modeling were used to assess the association between the number of drugs with all-cause 1-year mortality and overall survival. We adjusted for age, CCI, BMI, PSA, race, and prior treatment with docetaxel. Data were displayed using the Kaplan–Meier method. SAS 9.4 (SAS Inc., Cary NC) was used for analyses, and SPSS 28 and Microsoft Excel were used to create figures. All tests were two-sided. *p* values less than 0.05 were considered statistically significant. Confidence intervals that did not contain 1, the null value, were considered statistically significant. The data analyzed in this study are not publicly available due to security protocols and privacy regulations, but deidentified data are available upon request to the authors.

## 3. Results

### 3.1. Cohort Description

Between May 2011 and June 2017, 8855 Veterans who received treatment with abiraterone or enzalutamide for mCRPC were identified. The mean patient age was 74 years, and 25.7% of patients were Black (*n* = 2274). The median CCI was 3, which correlates to a 77% estimated 10-year survival in patients without metastatic cancer. A comparison of baseline patient characteristics is shown in Table 1. Among these 8855 Veterans, a median of 11 (6.16) unique medications and 6 (4.8) unique medication classes were filled in the year prior to treatment. The top 10 medications and medication classes are shown in Table 2.

### 3.2. Number of Medications Stratified by CCI, Age, and Race

**Figure 1:**
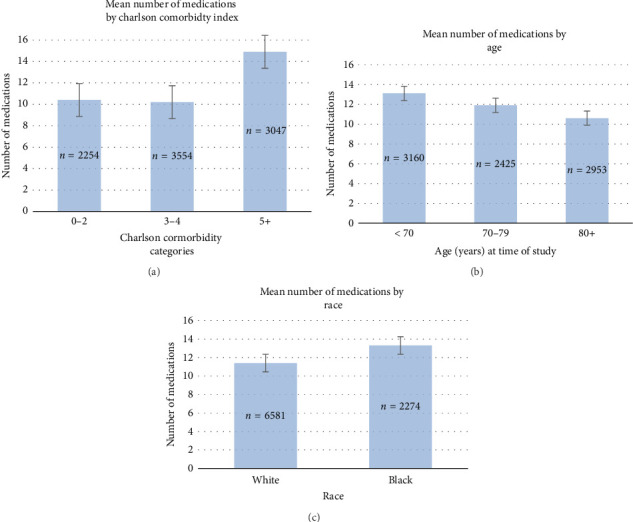
Number of medications prescribed by comorbidity, age, and race. Bar graphs showing medications prescribed increases with Charlson Comorbidity Index groupings (a) and black race (c) and decreases with age at time of study (b) in 8855 veterans. CCI 0–2 (90%–98% estimated 10-year survival), CCI 3-4 (53%–77% estimated 10-year survival), CCI 5+ (< 21% estimated 10-year survival).

The number of medications increased when progressing from a CCI of 3–4 to a CCI of 5+ as seen in Figure 1(a). Increasing age was associated with increased CCI across all age strata with a mean CCI of 3.7 in age < 70, 4.3 in age 70–79, and 4.6 in age 80+ (*p* < 0.001). Increasing age was associated with a decreased number of unique medications with a mean of 13.1 medications in age < 70, 11.9 in age 70–79, and 10.6 in age 80+ (*p* < 0.001), as seen in Figure 1(b). Black race was associated with an increased number of mean medications compared to White race (13.3 vs. 11.4, *p* < 0.001), as seen in Figure 1(c).

### 3.3. Age, CCI, and Number of Medications Are Independently Associated With Increased Mortality and Decreased Overall Survival

**Figure 2:**
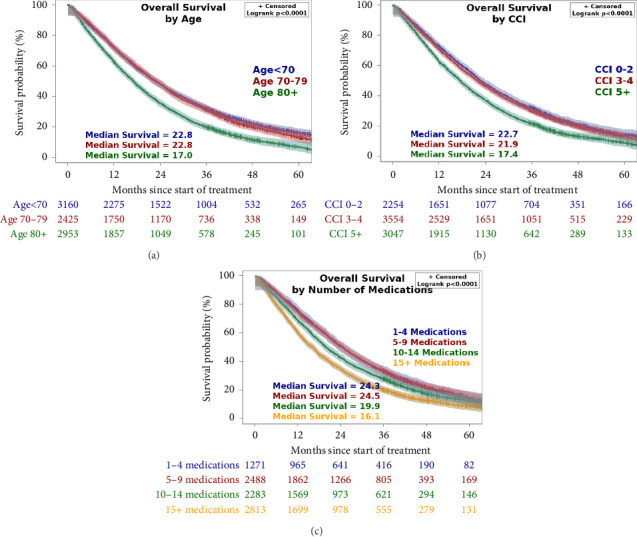
Overall survival by age, Charlson Comorbidity Index (CCI), and number of medications. Kaplan–Meier curves showing univariate overall survival in months by age group (a), Charlson Comorbidity Index group (b), and number of medications (c). + indicates censored data. Patient was removed from the study before experiencing death. Exact survival time is unknown.

Increasing age was associated with decreased survival with a median OS of 22.8 in age < 70, 22.8 in age 70–79, and 17.0 in age 80+ (*p* < 0.0001), as shown in Figure 2(a). Similarly, increasing CCI was associated with decreased survival with a median OS of 22.7 months in patients CCI 0–2, 21.9 months in CCI 3–4, and 17.4 months in CCI 5+ (*p* < 0.0001), as shown in Figure 2(b). For reference, there is a 98% estimated 10-year survival for a CCI score of 0, 96% for CCI 1, 90% for CCI 2, 77% for CCI 3, 53% for CCI 4%, and 21% for CCI 5. Additionally, the increasing number of medications was associated with decreased survival with a median OS of 24.3 months in patients on one to four medications, 24.5 months on five to nine medications, 19.9 months on 10–14 medications, and 16.1 months on 15+ medications (*p* < 0.0001), as shown in Figure 2(c).

To address whether medications might also be a useful clinical tool to predict survival in mCRPC patients, we then assessed whether the number of medications or the number of medication classes was independently associated with mortality. After adjusting for age, CCI, BMI, PSA, race, and prior treatment with docetaxel, both the number of medications and the number of medication classes were each associated with increased 1-year mortality with an adjusted OR (95% CI) of 1.03 (1.03, 1.04) for the number of medications and 1.08 (1.06, 1.11) for the number of medication classes. Both the number of medications and the number of medication classes were also associated with decreased OS with an adjusted hazard ratio (aHR) of 1.02 (1.01, 1.02) and 1.04 (1.03, 1.05).

**Figure 3:**
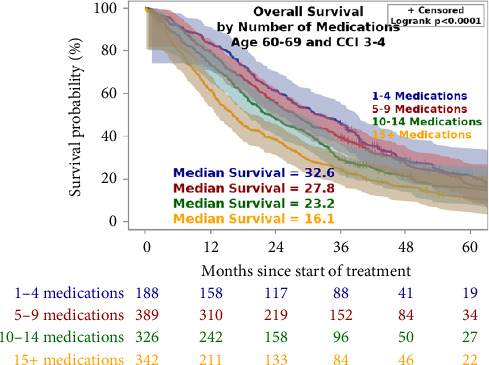
Overall survival within a subgroup of Veterans. Kaplan–Meier curves showing univariate overall survival in months in Veterans 60–69 years of age and CCI 3–4. + indicates censored data. Patient was removed from the study before experiencing death. Exact survival time is unknown.

Within a subgroup of patients with comparable age and CCI, an increased number of medications was associated with an increased risk of death. This subgroup was selected as it is the most common age of prostate cancer diagnosis and the most common CCI was 3 in our cohort of Veterans. As shown in Figure 3, we found a median OS of 32.6 months in patients on one to four medications, 27.8 months on five to nine medications, 23.2 months on 10–14 medications, and 16.1 months on 15+ medications. These data demonstrate that medications are more predictive of survival than age and CCI alone within this subset of patients.

### 3.4. The Association Between Medication Class and Overall Survival

**Figure 4:**
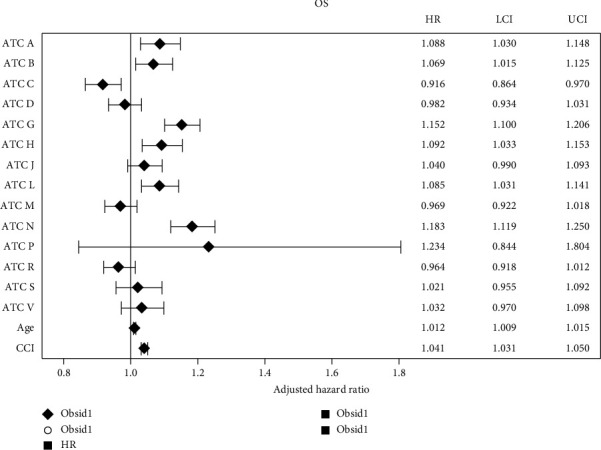
Overall survival by medication class. Forest plot showing overall survival based on ATC medication class. HR < 1 indicates decreased OS^∗^, HR > 1 increased OS^∗^, and HR = 1 no difference in survival. ^∗^Compared to Veterans not on medication in that Class A (alimentary tract and metabolism), Class B (blood and blood forming organs), Class C (cardiovascular), Class D (dermatological), Class G (genitourinary and sex hormones), Class H (systemic hormonal preparations), Class J (anti-infective for systemic use), Class L (antineoplastic and immunomodulating agents), Class M (musculoskeletal), Class N (nervous system), Class P (antiparasitic, insecticides, and repellents), Class R (respiratory), Class S (sensory organs), and Class V (various); LCI = lower confidence interval, UCI = upper confidence interval.

We also found that medication classes are predictive of survival. Treatment with medications in ATC N (nervous system) and ATC G (genitourinary and sex hormones) was associated with significantly worse overall survival compared to those not on these medication classes, aHR 1.18 (1.11, 1.25) and aHR 1.15 (1.10, 1.20), respectively. Additionally, ATC B (blood and blood-forming organs), aHR 1.06 (1.01, 1.12), ATC A (alimentary tract and metabolism), aHR 1.08 (1.03, 1.14), ATC L (antineoplastic and immunomodulating agents), aHR 1.08 (1.03, 1.14), and ATC H (systemic hormonal preparations), aHR 1.09 (1.03, 1.15), were associated with decreased overall survival. The only medication class that was associated with increased overall survival was ATC C (cardiovascular), aHR 0.91 (0.86, 0.97), as shown in Figure 4. ATC P (antiparasitic, insecticides, and repellents) is a rarely used prescribed drug class within this cohort, explaining the wide CI.

## 4. Discussion

In a large, nationwide cohort of Veterans with mCRPC, prescription medications serve as a marker of mortality risk and a surrogate for health status. As expected, increasing CCI was associated with a higher number of medications, as medicines are used to treat comorbid conditions. However, within subgroups of patients with comparable age and CCI, the increased number of medications was associated with the increased risk of death, indicating that the number of medications is more predictive and specific for the risk of death compared to CCI. Comorbidity indices that are based on ICD codes require accurate and complete documentation; however, underreporting or failure to code for comorbidities prevents these indices from being the most reliable tool to quantify disease. Prescription history is readily available, inclusive, and not dependent on manual coding or complex weighting. Thus, as our data demonstrate, using medications as a surrogate for traditional comorbidity indices may serve as a more accurate way to identify comorbid diseases and predict mortality.

Increasing age was associated with increased CCI. However, we found that the number of medications was inversely associated with age. These findings are contrary to the general population where polypharmacy in the elderly population is a growing problem [22–24]. This may be attributed to the increasing implementation of clinical pharmacists within the VA health system who are trained to review medications, provide medication counseling, and ensure that medications are being prescribed appropriately.

Another correlation we identified was the increased number of medications in Black patients compared to White patients. Given that U.S. Veterans have comprehensive healthcare, we do not believe these findings are due to unequal access to care. It is known that Black men have a higher incidence of prostate cancer, have a higher prostate cancer mortality, are diagnosed at a younger age [25–27], and have higher rates of comorbid diseases [28]. This population may benefit from more intensive management of comorbid diseases to address this disparity. By identifying patients at higher risk, providers can better determine which patients would benefit from closer follow-up, increased patient education, home health, and other supportive services. Additionally, more objectively determining risk may help guide treatment decisions and advance care planning discussions. Future studies should determine if these findings are replicated in the general population.

Specific medication classes were also prognostic for survival. Surprisingly, cardiovascular medications were associated with longer survival. This finding could be attributed to statins, a frequently prescribed medication within this class used to lower serum lipid levels. Statins have been shown to improve prognosis in mCRPC treated with enzalutamide or abiraterone through the reduction of cholesterol and downstream androgens necessary to drive prostate cancer growth [29, 30]. Future studies should focus on the impact of other cardiovascular medications on survival, such as antihypertensives or diuretics, which may indicate the severity of conditions such as heart failure. Medications within ATC G (genitourinary and sex hormones) and ATC N (nervous system) were the most strongly associated with decreased survival. This could be because medications within these classes are more so used to alleviate symptoms rather than treat a specific comorbidity and are likely indicators of general frailty and poorly controlled disease. Some examples include selective estrogen receptor modulators used to treat osteoporosis, antimuscarinics for overactive bladder, scopolamine for nausea, and analgesics for pain. While analysis of individual medications was outside the scope of this study, it may be beneficial for future studies to evaluate if specific medications or combinations of medications are linked to decreased survival.

### 4.1. Limitations

The Veteran population is a unique cohort of patients who generally have access to comprehensive healthcare, including improved prescription drug coverage, access to specialists, and access to mental health resources. Veterans may also differ in comorbidity prevalence and socioeconomic status from the general population. Similar to other retrospective studies, causality between the number of medications and mortality cannot be determined due to unknown confounders. Polypharmacy remains an important public health concern, which is not limited to Veterans. Polypharmacy, which is associated with an increased risk of drug–drug interactions, falls, cognitive impairment, hospitalizations, and mortality, is a potential confounder. Unfortunately, it is impossible to control physician prescribing practices in retrospective studies, which can lead to misleading associations. We must also assume medications are being prescribed appropriately, which is another potential confounder. Additionally, the number of medications was calculated from VHA prescriptions, so if patients received any medications from outside of the VHA, they would not have been included in this study. To limit misrepresentation of comorbidities, any Veteran with zero dispensed prescription medications was excluded. Furthermore, adherence to prescription medicines is not able to be assessed in this retrospective study and is likely associated with outcomes. We attempted to decrease the impact of nonadherence by only evaluating prescription medications that were dispensed from the pharmacy.

### 4.2. Strengths and Future Directions

This study has several strengths, such as the use of a large, nationally representative sample of U.S. Veterans. Most Veterans who choose to receive care in the VHA receive most, if not all, of their care within the VHA system, which allows for a comprehensive dataset with low missingness. The ATC coding system is widely utilized in many countries and only requires the availability of prescription data, making it easily applicable in all healthcare contexts at the population level.

Future research should verify whether this pattern of findings we observed is consistent across the non-Veteran population or other malignancies. Future studies may choose to delineate which medications are prescribed for prevention, comorbid diseases, or for the management of side effects, which may provide additional prognostic data. Additionally, future work may choose to implement patient self-report questionnaires, pharmacy refill records, or therapeutic drug monitoring to limit the impact of nonadherence on data outcomes.

Improving our understanding of comorbid disease has several clinical applications. Patients on certain medications linked to higher mortality may benefit from the integration of clinical pharmacists to decrease polypharmacy, adverse drug reactions, and potential drug–drug interactions. Patients with a higher burden of disease would benefit from utilization of additional healthcare services such as home nursing care or therapy to help prevent hospitalizations and decrease the costs of medical care. By objectively assessing the risk associated with comorbid diseases, clinicians are likely to achieve more accurate mortality prognostication to facilitate advance care planning and preparation for end of life. There are also several treatment implications associated with comorbid diseases. Several cancer therapeutics are contraindicated or need to be dose-adjusted based on organ dysfunction. Patients who are more likely to die from a comorbid condition may not benefit from aggressive cancer treatments, which could potentially cause more harm than good and decrease quality of life. While these are only some of the important clinical implications, future research should focus on implementing these findings into treatment protocols and healthcare policies for patients with cancer.

## 5. Conclusion

In conclusion, this nationwide study of U.S. Veterans identified a relationship between the number of prescription medications and outcomes in patients undergoing treatment for mCRPC, even after accounting for important covariates including age, CCI, BMI, PSA, and race. Moreover, these data suggest that the number of medications can be used to further stratify a subset of mCRPC patients aged 60–69 years with an intermediate CCI of 3–4 and more accurately predict survival in this population. With the approval of new therapies for treatment of advanced prostate cancer, patients are living longer with this disease. An understanding of risk factors and predictors of survival through assessment of prescription medications may improve the outcomes of treatment in this patient population.

## Figures and Tables

**Table 1:** Demographics and clinical characteristics.

Patient characteristics	*N* = 8855
Age (years)	
Mean (SD)	74 (9.36)
Median (IQR)	74 (67.82)
< 70	3160 (37.01)
70–79	2425 (28.40)
80+	2953 (34.59)
Number of medications	
Mean (SD)	11.85 (7.03)
Median (IQR)	11 (6.16)
1–4	1271 (14.35)
5–9	2488 (28.10)
10–14	2283 (25.78)
15+	2813 (31.77)
Number of medication classes (based on Anatomical Therapeutic Chemical Classification System)	
Mean (SD)	5.99 (2.54)
Median (IQR)	6 (4.8)
Charlson Comorbidity Index	
Mean (SD)	4.23 (2.70)
Median (IQR)	3 (2.6) −> max = 18
0–2 (90%–98% estimated 10-year survival)	2254 (25.45)
3-4 (53%–77% estimated 10-year survival)	3554 (40.14)
5+ (< 21% estimated 10-year survival)	3047 (34.41)
Race	
Black	2274 (25.68)
White	6581 (74.32)
PSA (ng/mL)	
0–4	737 (8.32)
4–10	940 (10.62)
10–20	1177 (13.29)
20–50	1851 (20.90)
50–100	1461 (16.50)
100–200	1021 (11.53)
200+	1668 (18.84)
Docetaxel prior to treatment	
No	6874 (77.63)
Yes	1981 (22.37)
BMI (kg/m^2^)	
< 18.5	208 (2.35)
18.5–24.9	2438 (27.53)
25–29.9	3198 (36.12)
30+	3011 (34.00)

*Note:* A table displaying the demographics and clinical characteristics of the cohort of Veterans with mCRPC treated with abiraterone or enzalutamide between 2011 and 2017 examined in this study.

Abbreviations: BMI, body mass index; IQR, interquartile range; SD, standard deviation.

**Table 2:** Most commonly prescribed medications.

Medication	Medication class (based on the Anatomical Therapeutic Chemical Classification System)	Example indications for use
Bicalutamide (*n* = 5185)	ATC L (antineoplastic and immunomodulating agents)	Metastatic prostate cancer
Acetaminophen (*n* = 3784)	ATC N (nervous system)	Pain, fever
Lisinopril (*n* = 2673)	ATC C (cardiovascular system)	Antihypertensive
Omeprazole (*n* = 2646)	ATC A (alimentary tract and metabolism)	Indigestion, heartburn, acid reflux, ulcer prevention
Prednisone (*n* = 2621)	ATC H (systemic hormonal preparations)	Anti-inflammatory, immunosuppressive
Hydrocodone (*n* = 2595)	ATC N (nervous system)	Pain
Docusate (*n* = 2500)	ATC A (alimentary tract and metabolism)	Stool softener
Tamsulosin (*n* = 2241)	ATC G (genitourinary and sex hormones)	Benign prostatic hyperplasia
Simvastatin (*n* = 2085)	ATC C (cardiovascular system)	Lower cholesterol
Amlodipine (*n* = 2004)	ATC C (cardiovascular system)	Antihypertensive

*Note:* Table displaying the 10 most commonly prescribed medications by name, medication class, and the primary indications for use in this cohort of Veterans.

## Data Availability

The data analyzed in this study are not publicly available due to security protocols and privacy regulations, but deidentified data are available upon request to the authors.

## References

[1] Ng K., Smith S., Shamash J. (2020). Metastatic Hormone-Sensitive Prostate Cancer (mHSPC): Advances and Treatment Strategies in the First-Line Setting. *Oncology and Therapy*.

[2] de Bono J. S., Logothetis C. J., Molina A. (2011). Abiraterone and Increased Survival in Metastatic Prostate Cancer. *New England Journal of Medicine*.

[3] Scher H. I., Fizazi K., Saad F. (2012). Increased Survival With Enzalutamide in Prostate Cancer After Chemotherapy. *New England Journal of Medicine*.

[4] Grönberg H. (2003). Prostate Cancer Epidemiology. *The Lancet*.

[5] Ncis (2024). Cancer Stat Facts: Prostate Cancer. https://seer.cancer.gov/statfacts/html/prost.html.

[6] Schoen M. W., Carson K. R., Eisen S. A. (2022). Survival of Veterans Treated With Enzalutamide and Abiraterone for Metastatic Castrate Resistant Prostate Cancer Based on Comorbid Diseases. *Prostate Cancer and Prostatic Diseases*.

[7] Charlson M. E., Pompei P., Ales K. L., MacKenzie C. R. (1987). A New Method of Classifying Prognostic Comorbidity in Longitudinal Studies: Development and Validation. *Journal of Chronic Diseases*.

[8] Elixhauser A., Steiner C., Harris D. R., Coffey R. M. (1998). Comorbidity Measures for Use With Administrative Data. *Medical Care*.

[9] Quan H., Sundararajan V., Halfon P. (2005). Coding Algorithms for Defining Comorbidities in ICD-9-CM and ICD-10 Administrative Data. *Medical Care*.

[10] Lloyd S. S. (1985). Physician and Coding Errors in Patient Records. *JAMA, the Journal of the American Medical Association*.

[11] Horsky J., Drucker E. A., Ramelson H. Z. (2017). Accuracy and Completeness of Clinical Coding Using ICD-10 for Ambulatory Visits. *AMIA Annual Symposium proceedings AMIA Symposium*.

[12] Wilchesky M., Tamblyn R. M., Huang A. (2004). Validation of Diagnostic Codes Within Medical Services Claims. *Journal of Clinical Epidemiology*.

[13] Von Korff M., Wagner E. H., Saunders K. (1992). A Chronic Disease Score From Automated Pharmacy Data. *Journal of Clinical Epidemiology*.

[14] Clark D. O., Korff M. V., Saunders K., Balugh W. M., Simon G. E. (1995). A Chronic Disease Score with Empirically Derived Weights. *Medical Care*.

[15] Malone D. C., Billups S. J., Valuck R. J., Carter B. L. (1999). Development of a Chronic Disease Indicator Score Using a Veterans Affairs Medical Center Medication Database. *Journal of Clinical Epidemiology*.

[16] Fishman P. A., Goodman M. J., Hornbrook M. C., Meenan R. T., Bachman D. J., O’Keeffe Rosetti M. C. (2003). Risk Adjustment Using Automated Ambulatory Pharmacy Data. *Medical Care*.

[17] Gilmer T., Kronick R., Fishman P., Ganiats T. G. (2001). The Medicaid Rx Model. *Medical Care*.

[18] George J., Phun Y.-T., Bailey M. J., Kong D. C. M., Stewart K. (2004). Development and Validation of the Medication Regimen Complexity Index. *The Annals of Pharmacotherapy*.

[19] Robusto F., Lepore V., D’Ettorre A. (2016). The Drug Derived Complexity Index (DDCI) Predicts Mortality, Unplanned Hospitalization and Hospital Readmissions at the Population Level. *PLoS One*.

[20] Gedeborg R., Garmo H., Robinson D., Stattin P. (2020). Prescription-Based Prediction of Baseline Mortality Risk Among Older Men. *PLoS One*.

[21] Romano P. S., Roos L. L., Jollis J. G. (1993). Presentation Adapting a Clinical Comorbidity Index for Use With ICD-9-CM Administrative Data: Differing Perspectives. *Journal of Clinical Epidemiology*.

[22] Charlesworth C. J., Smit E., Lee D. S. H., Alramadhan F., Odden M. C. (2015). Polypharmacy Among Adults Aged 65 Years and Older in the United States: 1988–2010. *The Journals of Gerontology Series A: Biological Sciences and Medical Sciences*.

[23] Hajjar E. R., Cafiero A. C., Hanlon J. T. (2007). Polypharmacy in Elderly Patients. *The American Journal of Geriatric Pharmacotherapy*.

[24] Morin L., Johnell K., Laroche M. L., Fastbom J., Wastesson J. W. (2018). The Epidemiology of Polypharmacy in Older Adults: Register-Based Prospective Cohort Study. *Clinical Epidemiology*.

[25] Hinata N., Fujisawa M. (2022). Racial Differences in Prostate Cancer Characteristics and Cancer-specific Mortality: An Overview. *The World Journal of Men’s Health*.

[26] Robbins H. A., Engels E. A., Pfeiffer R. M., Shiels M. S. (2015). Age at Cancer Diagnosis for Blacks Compared With Whites in the United States. *Journal of the National Cancer Institute*.

[27] Karami S., Young H. A., Henson D. E. (2007). Earlier Age at Diagnosis: Another Dimension in Cancer Disparity?. *Cancer Detection and Prevention*.

[28] Freeman V. L., Durazo-Arvizu R., Keys L. C., Johnson M. P., Schafernak K., Patel V. K. (2004). Racial Differences in Survival Among Men With Prostate Cancer and Comorbidity at Time of Diagnosis. *American Journal of Public Health*.

[29] Harshman L. C., Werner L., Tripathi A. (2017). The Impact of Statin Use on the Efficacy of Abiraterone Acetate in Patients with Castration-Resistant Prostate Cancer. *The Prostate*.

[30] Yang H., Pang L., Hu X. (2020). The Effect of Statins on Advanced Prostate Cancer Patients With Androgen Deprivation Therapy or Abiraterone/Enzalutamide: A Systematic Review and Meta-Analysis. *Journal of Clinical Pharmacy and Therapeutics*.

